# A statistical tool for comparing seasonal ILI surveillance data

**DOI:** 10.1038/s41598-018-38292-x

**Published:** 2019-02-05

**Authors:** René Ferland, Sorana Froda

**Affiliations:** Département de mathématiques, UQAM, C.P. 8888, succursale centre-ville, Montréal, Québec H3C 3P8 Canada

## Abstract

In this paper, we consider the yearly influenza epidemic, as reflected in the seasonal surveillance data compiled by the CDC (Center for Disease Control and Prevention, USA) and we explore a new methodology for comparing specific features of these data. In particular, we focus on the ten HHS (Health and Human Services) regions, and how the incidence data evolves in these regions. In order to perform the comparisons, we consider the relative distribution of weekly new cases over one season and replace the crude data with predicted values. These predictions are obtained after fitting a negative binomial regression model that controls for important covariates. The prediction is computed on a ‘generic’ set of covariate values that takes into account the relative size (population wise) of the regions to be compared. The main results are presented in graphical form, that quickly emphasizes relevant features of the seasonal data and facilitates the comparisons.

## Introduction

The yearly flu season in North America (defined as starting in the 40th week of each year, i.e. the first full week in October) has an important yearly impact, both in morbidity and mortality. Since October 1997 the Center for Disease Control and Prevention (in short CDC) has systematically collected data on the flu season through various channels, one being the weekly new cases of influenza like illness (abbreviated as ILI) that are reported by the U.S. Outpatient Influenza-like Illness Surveillance Network (abbreviated ILINet). As detailed in^1^, these are volunteer outpatient healthcare providers (according to^1^, in the past few years, there were more than 2800 such practices); the enrollment covers all 50 states, Puerto Rico, the District of Columbia, and the U.S. Virgin Islands. Every Tuesday, these health practitioners report the total number of patients seen the previous week (i.e. Sunday to Saturday) for any reason (‘visits’) and how many patients had influenza-like illness (ILI). In^1^ the ILI is defined as fever (temperature of at least 100 deg. F or 37.8 deg. C) and a cough and/or a sore throat, when there is no other known cause for the condition. The providers also indicate the age group (0–4 years, 5–24 years, 25–49 years, 50–64 years, and ≥65 years) of each patient. Such surveillance data are made public, lately through the *Flu View* tool posted on the CDC website. On this site, one can access national data and two types of regional data, namely: as distributed in ten HHS (Health and Human Services) regions or in nine census divisions. In this paper, we consider the ten HHS regions (the full list is available as Supplementary Information). During the first four seasons, i.e. from 1997–1998 to 2000–2001, the CDC posted the data for the first 33 weeks of the flu season only (i.e. from October till end of May). The definition of age groups slightly changed over time as well, as before the 2009–2010 season there were only 4 age groups (0–4 years, 5–24 years, 25–64 years, and ≥65 years) but this has no impact on the present analysis.

Given the nature of the surveillance data, the CDC is noting that it can be inappropriate to compare (across regions or years) either the total number of new cases or their incidence among visits. One such reason for rendering comparisons difficult is that the type of participating health practices can vary across regions, e.g. the proportion of pediatric ones can be more important in some areas. When comparing years, the difficulty comes from the fact that the number of participating health providers has steadily increased over time, and thus there is also an increasing trend in the reported cases. One could remedy this by working with incidence rates; on the other hand, even in the same season these incidence rates are still quite variable (an example of such crude data is given in the last section). In its published graphs (national and regional) the CDC is weighting the observed incidence rates by a factor that takes into account the population size but the final values are not qualitatively different from the crude data.

This being said, there has been a genuine interest in the literature to assess the evolution of flu epidemics over time and space, and perform various comparisons, across regions, cities, and even countries. If we restrict our attention to the flu season, i.e. we do not consider the pandemic years, we can note two major research directions: either to compare the data collected by health agencies (viewed as benchmark or golden standard) with the ones produced by Google, Twitter or other social media as in^2–5^ or, indeed, to compare the seasonal evolution of epidemics as reported by various national or regional health agencies, as in^6–8^. These last authors are mainly preoccupied with the time synchrony of epidemics and have used time series fitting techniques. Moreover, some authors, e.g.^7^, deal with mortality data rather than disease incidence. Another area of active research is to explore alternative ways of performing influenza surveillance, by relating traditional influenza surveillance systems to other measurement methods, for example the total number of specific medication prescriptions, as in^9^ or^10^.

In this paper, we take a different view from the above authors as we propose to treat time as a categorical variable: we consider $k=33$ classes (first 33 weeks of the flu season, i.e. from the first week in October until the last week in May). This choice is justified as these 33 weeks are reported in all seasons since 1997 and in every season most flu cases are declared before May. Further, we change the focus of the analysis, as we consider comparing the relative distribution of the weekly total number of new cases, or how the seasonal new cases are spread among the weeks of a given flu season. In other words, this comes to dividing the weekly number of new cases by the total case count over that season and comparing the resulting weekly proportions. One could compute these proportions based on the crude data but the CDC expresses reservations and adds cautionary notes on performing comparisons based on this type of crude data. To illustrate this point, assume that we want to compare the proportion of ILI cases in two regions, during the same week, and in each region we deal with two age groups. Further, assume that the two age groups are distributed as $({r}_{1},1-{r}_{1})$ in Region 1 and $({r}_{2},1-{r}_{2})$ in Region 2; for the sake of the argument, let the first age group be “young” (children) and more prone to falling ill. Further, assume that the proportion of cases in each age group is the same in both regions, with *p* the proportion in age group “young”, *q* in the other age group, and $p > q$. Then, the difference in the regional proportion of cases, *π*_1_, *π*_2_, depends only on the age distribution inside each region, since:

1 $${\pi }_{1}=p\cdot {r}_{1}+q\cdot (1-{r}_{1})=(p-q)\cdot {r}_{1}+q < (p-q)\cdot {r}_{2}+q={\pi }_{2},\,{\rm{if}}\,{r}_{1} < {r}_{2},$$

2 $${\pi }_{1}=p\cdot {r}_{1}+q\cdot (1-{r}_{1})=(p-q)\cdot {r}_{1}+q=(p-q)\cdot {r}_{2}+q={\pi }_{2},\,{\rm{if}}\,{r}_{1}={r}_{2}.$$

Thus, in equation (1) the proportion *π*_2_ in Region 2 is higher due to the over representation in Region 2 of the age group that has a higher proportion *p* of cases. This over representation can be due to factors related to the disease but also to the way the data are collected, which is one of the caveats noted by the CDC. Indeed, if we base the analysis on crude proportions the following can happen: in one region there could be more pediatric participating practices and thus ILI cases that are children (age group “young”) could be over represented in this region and convey a “false” impression, based on an inequality like the one in equation (1). Of course, all this is a conceptual argument that can be applied when considering other factors.

Therefore, in order to address such issues, we propose to compare predicted proportions of weekly new cases that are adjusted for important explanatory variables that control for the time trend and some heterogeneities in the data. In this type of comparison, the impact of the regional fluctuations in the explanatory variables is controlled. Indeed, if we consider two estimated regional functions ${\hat{\pi }}_{j}={f}_{j}(r,u,v,\ldots ),\,j=1,2$ (where *r*, *u*, *v*, etc are explanatory variables) and evaluate them at the same values of these variables (in particular the proportion *r* defined above), then the inequality ${\hat{\pi }}_{1} < {\hat{\pi }}_{2}$ is not determined by the specific value of *r* (or *u*, *v* etc) but mainly reflects the difference in the flu progress between regions. The mathematics of how this is achieved in the generalized linear approach considered here (see next section) is too complex to be summarized analytically but one can give an elementary illustration on the difference between crude and predicted values in the case of simple linear regression (weighted or unweighted): assume that, for $i=1,\ldots ,n,$ one observes $({x}_{i},{y}_{i})$ in Region 1 and $(\lambda \cdot {x}_{i},{y}_{i}),\lambda  > 0$ in Region 2, and consider a linear fit $\hat{y}={a}_{j}+{b}_{j}x,j=1,2,$ one for each region. In this case, the intercepts ${a}_{1}={a}_{2}$ but the slopes differ and satisfy ${b}_{2}={b}_{1}/\lambda$. Hence, the predictions for fixed values of *x* are quite different in the two regions, although the observed ${y}_{i},i=1,\ldots ,n$ values are identical. As for the chosen explanatory variables in our application, we had to resort to the available information; we decided to take into account the total volume of visits to the health provider (that accounts for the yearly fluctuation), the number of participating practices (in order to account for the time trend over seasons), as well as the reported ILI cases that are in the 0 to 4 years age group. This information is readily available (i.e. in the public domain) and plays a crucial role in the seasonal distribution of new flu cases.

The details of our proposed comparison tool are given in the next section, while the difference between our approach and a comparison of crude proportions is illustrated in the last section.

## Method: Main Idea

**Figure 1:**
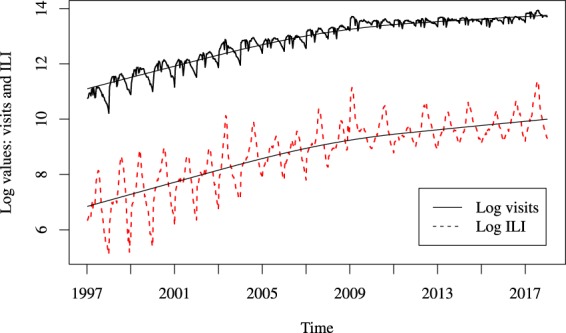
Trend of reported visits and ILI cases over 21 years. Logarithms of number of visits (full line) and number of flu cases (dotted line) and their trend, in 21 flu seasons (33 weeks per season), from October 1997 until May 2018.

The outpatient ILI data is based on reports made by the participating medical practices, which, at the national USA level, increased steadily from about 200 to more than 2500 between 1997 and the present day. Therefore, over time, there was a net increase in the reported number of visits and, in parallel, of reported cases; in Fig. 1 we represent as function of time both the reported number of visits and the reported number of flu cases, ILI. The data are in logarithmic scale and a smoother was used to indicate the trend.

In order to find a way of comparing the relative regional distribution of incident cases over one season we propose the following methodology that takes into account this time trend, among other:

- fit a regression model based on the regional data, collected from 1997 till the present; this gives 10 regional prediction equations;
- create specific ‘generic’ data sets, one for each region and each season, based on the same weekly distribution in a particular season;
- apply the regional prediction equation to the corresponding generic data set and compare the resulting regional predictions.

In the sequel, we explain in detail each step.

**Step a**. We start by fitting an appropriate regression model to the incidence rate of ILI cases, in each HHS region; this rate is the total of newly diagnosed ILI cases divided by the number of visits in a given week of the flu season. Thus, the main explanatory variable is the week (viewed as a categorical variable) and we propose to adjust the fitting by controlling for two other explanatory variables: the total number of participating health providers (as a proxy for the variation over time) and the total of ILI patients who are in the 0–4 age group (as a proxy for the number of pediatric practices among the surveillance clinics). An equivalent formulation is to regress the total number of cases on three explanatory variables, with the number of visits added to the previous two regressors. More precisely: we deal with count data (new ILI cases in a specific week) and we propose to fit a negative binomial regression model, which is a standard approach in cases where over dispersion is present; see e.g.^11^. In such a count model, the link function is $\mathrm{log}\,E[Y]$, where in our context *Y* is the number of new ILI cases in a given region in a specific week (for instance, in our data analysis, there were $n=660$ weeks in total).

Thus, each regional model can be written as follows (two equivalent equations, we omit the subscript for region):

3 $$\mathrm{log}\,E[{Y}_{j}]=\,\mathrm{log}({V}_{j})+{\beta }_{0}+{\beta }_{1}{M}_{j}+{\beta }_{2}{A}_{j}+{\beta }_{3}{M}_{j}{A}_{j}+\sum _{k=2}^{33}\,{\gamma }_{k}{I}_{jk}$$

4 $$\iff \,\mathrm{log}\,E[\frac{{Y}_{j}}{{V}_{j}}]={\beta }_{0}+{\beta }_{1}{M}_{j}+{\beta }_{2}{A}_{j}+{\beta }_{3}{M}_{j}{A}_{j}+\sum _{k=2}^{33}\,{\gamma }_{k}{I}_{jk},$$

where, for $j=1,\ldots ,n$ (*n* weeks in all): *Y*_*j*_ is the total number of ILI incident cases, *V*_*j*_ is the total number of visits, *M*_*j*_ is the number of participating medical practices, *A*_*j*_ is the number of ILI cases aged 0 to 4, and ${I}_{jk},k=2,3,\ldots 33$ are the indicator variables corresponding to 32 weeks of one season (2nd to 33d); the first week of the season is the base category. In the formulation given in equation (3), where we regress the total number of incident cases (in logarithmic scale), log(*V*_*j*_) is the offset. We suspected that the impact of the number of health providers could be affected by the volume of participating pediatricians and therefore introduced the interaction term between the variable that serves as a proxy for the latter (ILI cases that are in the 0–4 age group) and the number of participating health practices. Our guess was confirmed by the statistical analysis as the interaction term improved the fit substantially.

**Step b**. As our aim is to compare the progress of the flu epidemic over a given season across regions, we suggest to plot (or tabulate) the predictions given by the model(s) obtained above (in Step a.) as applied to a common data set of explanatory variables. The challenge is in defining such a common data set, as the size of the regions varies a lot; for instance, inserting the observed national values in the regional equation (3) does not make much sense, as in some cases the national values can be twenty times bigger than the original regional values. Therefore, we propose the following approach: for a given season, compute the national relative distribution (by week, out of 33 weeks) of each explanatory variable and further create ‘generic’ regional data by applying this relative distribution to the regional total (of each variable) over one season. For illustration, let the season be fixed and let ${M}_{k}^{N}$ be the national value and ${M}_{k}^{R}$ be the observed regional value, during week *k* of that season, $k=1,2,\ldots ,33$. Then, the generic value ${M}_{k}^{g,R}$ of the explanatory variable *M* in region *R* is computed as follows:

$${M}_{k}^{g,R}=(\sum _{\ell =1}^{33}\,{M}_{\ell }^{R})\cdot \frac{{M}_{k}^{N}}{{\sum }_{\ell =1}^{33}\,{M}_{\ell }^{N}},\,k=1,2,\ldots ,33.$$

This way, the relative distribution over one specific season of the ‘generic’ values ${M}_{k}^{g,R},k=1,2,\ldots ,33$, is the same in all regions, while their order of magnitude corresponds to the one of the original data in each region.

**Step c**. Once the ‘generic’ regional data set is created, insert it in equation (3) (one such equation per region) which gives the corresponding predicted number of incident cases per week, $\hat{Y}$. The seasonal predicted values ${\hat{Y}}_{1},{\hat{Y}}_{2},\ldots ,{\hat{Y}}_{33}$ are further divided by their sum and the resulting proportions ${\hat{p}}_{1},{\hat{p}}_{2},\ldots ,{\hat{p}}_{33}$ can be graphically visualized or otherwise compared.

A final important remark is that in such an approach one cannot expect to have curves lying above one another for the whole time period, as they correspond to a relative distribution of new (incident) cases among the 33 weeks of the season. For example, if at the start of the season the proportion of cases is higher in location A than in location B, then later in the season this proportion is necessarily lower in location A than in location B, as the seasonal proportions sum up to one in both locations.

## Results

Our analysis is based on the data from $n=660$ weeks, chosen as follows: out of 21 seasons, we eliminated the pandemic flu season (2009–2010); also, to gain power, we excluded the category “week 53”, as there was such a week only three times in 21 years. Further, we applied the method described in the previous Section: we performed the negative binomial fitting based on $n=660$ weeks and further we computed predictions. To illustrate our methodology, we focus on some selected seasons. As can be noted in Fig. 1, one can divide the observed seasons 1997–1998 to 2017–2018 into three *regimes* (we exclude the pandemic year 2009–2010), whether by volume of medical visits or by number of new cases which mirror them: low, medium, high. These regimes do not correspond to an increase in the number of cases, but are mainly a reflection of the fact that the number of participating health providers (and therefore the number of visits) has steadily increased over time. Thus, although our fitting method takes into account this crucial factor, we found interesting to consider a typical season in each such regime, and illustrate our method by considering the following three selected seasons: Season 5 (2001–2002, ‘low’ regime), Season 10 (2006–2007, ‘medium’ regime), and Season 16 (2012–2013, ‘high’ regime). Further, it must be noted that the population of the ten HHS regions varies a lot, as some are much more populated than others. This way we identified three region sizes: small (less than 5% of the USA population), medium (between 9.5% and 12.8% of the USA population), large (more than 15.5% of the USA population).

So, in order to facilitate the graphical comparison, we divided the ten HHS regions in three classes according to two criteria:

- geographical location: East (regions 1 to 4), Central (regions 5, 6, 7), West (regions 8, 9, 10);
- size of regions, as described above: small (regions 1, 7, 8, 10), medium (regions 2, 3, 6), large (regions 4, 5, 9).

Further, we compared the regions inside each class.

**Figure 2:**
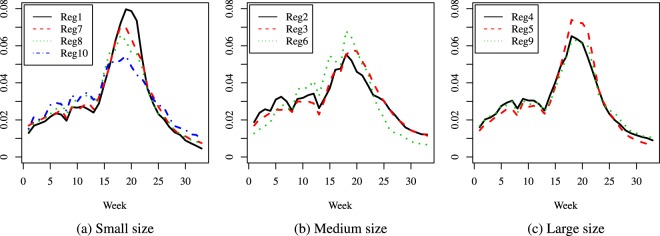
Season 5: comparison of predicted relative proportions, regions grouped by population size.

**Figure 3:**
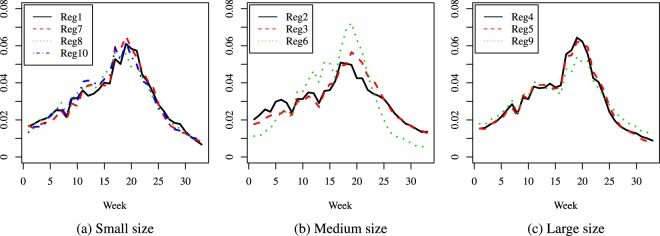
Season 10: comparison of predicted relative proportions, regions grouped by population size.

**Figure 4:**
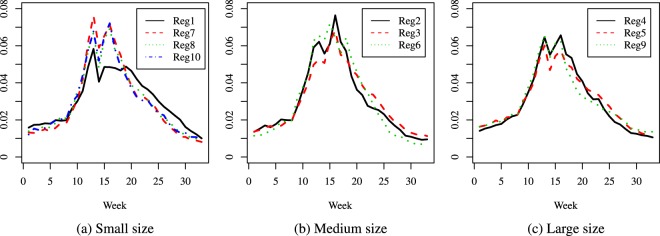
Season 16: comparison of predicted relative proportions, regions grouped by population size.

**Figure 5:**
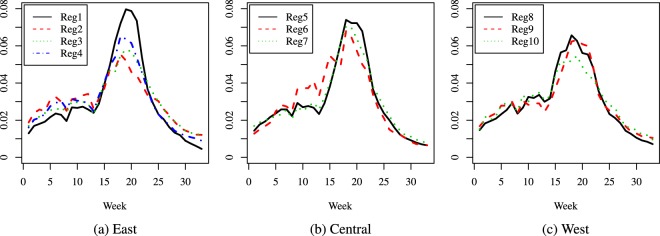
Season 5: comparison of predicted relative proportions, regions grouped by geographical location.

**Figure 6:**
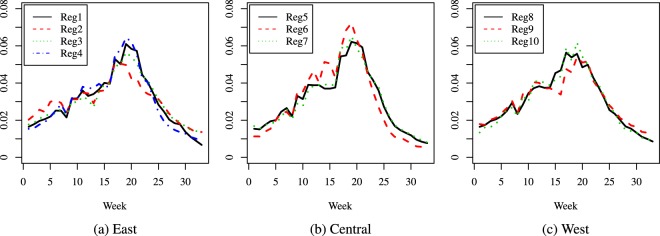
Season 10: comparison of predicted relative proportions, regions grouped by geographical location.

**Figure 7:**
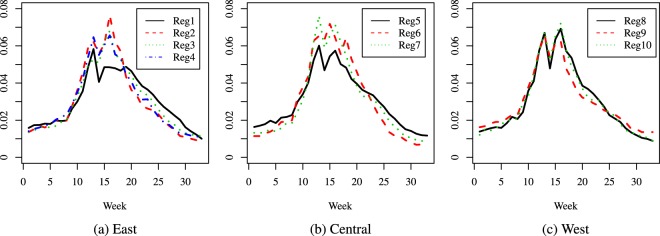
Season 16: comparison of predicted relative proportions, regions grouped by geographical location.

The results are presented as curves as follows: Figs 2, 3 and 4 give the comparisons where the regions were grouped by population size, while Figs 5, 6 and 7 give the comparisons where the regions were grouped by geographical location.

How can one read these results? Given the way the curves are created, we address the following matter: to compare the seasonal progress of the epidemic across regions, when the weekly distribution of relevant explanatory variables is the same (in other words, under identical conditions for all regions). Thus, the procedure renders the regions much more comparable and performs a certain amount of smoothing, without hiding essential features, like the bimodal character in Season 16. This is quite different from a multi-modality that may be an artificial by-product of the way the cases are reported.

Finally, it is worth noting that^12^ have also considered the issue of rendering epidemic curves comparable; their research proposes a way of aligning epidemic curves corresponding to laboratory tests data, in order to compare influenza seasons.

## Discussion and Conclusion

To summarize, the paper introduces two novel ideas in comparing the dynamics of an epidemic season: a first one is to compare relative distributions rather than crude incidence rates and the second one is to resort to predicted rather than crude data values. Our focus is mainly methodological, and our specific illustrations indicate that the method can highlight features like the size of the difference between the number of new cases at the height of the flu season and at its beginning or end, and distinguish regions according to such features: e.g., in some areas the epidemic has a very distinct peak, while in others it is more flat and cases are more evenly spread over the season; also, the distribution of cases can be more skewed to the right (end of season) or left (beginning of season). Another important finding is the different behavior across seasons. Given the seasonal aspect, in our comparisons we decided to group the regions according to their geographical location but we found interesting to take into account their population size as well, given the huge differences among regions; this led to a second grouping. In the medium size group, we observe a combined effect of these two factors (size and location): region 6 is consistently different from the other two, and it turns out that its location is in the Central part of the U.S.A., while the other two medium size regions are on the East Coast. In contrast, the large size category exhibits homogeneous dynamics. This being said, other groupings could be considered depending on the focus and interest of each practitioner. Also, note that the same methodology (for comparing predictions) can be applied to mortality or hospitalizations data.

**Figure 8:**
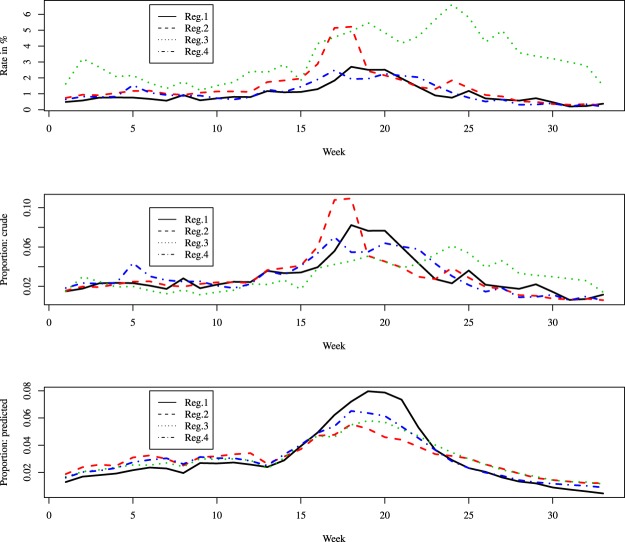
Three methods for comparing flu epidemics (season 5, East): observed incidence rates (top panel), proportions of observed incident cases by week (middle panel), proportions of predicted incident cases by week (bottom panel).

It can be instructive to contrast the present method to a comparison of the crude data. For illustration, we considered the four regions in the geographical location ‘East’ and the Season 5 (2001–2002) and analyzed them in three different ways; see Fig. 8. In the top panel of Fig. 8 there are the observed incidence rates (which is similar to what is reported by the CDC), while the other two panels represent seasonal proportions of observed ILI new cases (middle panel) and predicted ILI new cases (bottom panel). We can note big differences among the three approaches.

Indeed, in the middle panel we compute the relative distribution of the observed cases which corresponds in spirit to the proposed fitting method (proportion of new cases per week, among all 33 weeks); thus, unlike the top panel, in this middle panel the curves are better aligned; moreover, they necessarily have a similar order of magnitude, as they are on the same scale, and represent values from 0 to 1. Still, even after taking relative proportions, the curves in the middle panel (observed) and bottom panel (predicted) remain quite different. In the bottom panel (predicted values) Regions 3 and 4 have very similar behaviors (unlike what is seen in the middle panel), all three curves peak at about the same time (week 20 or mid-February), and the peak of Region 1 is higher than the one in the other three regions; this makes sense as Region 1 comprises New England. An interpretation of such a result is that in Region 1 there is a more pronounced peak in mid-season, while in the other regions the proportions of new cases evolve in a more even way. Applying this comparison methodology at local levels (counties, e.g.) may prove useful in planning health resources at specific times of the year. As noted above, in other seasons (like season 16 for example) the predictions can have a much more jagged aspect.

Another comment is on the variables that were retained in equation (3): our main concern was to avoid over-fitting, given the large number of degrees of freedom corresponding to the week factor. This being said, the emphasis of the paper is on the comparison methodology, and not on proposing the best model for this kind of data; moreover, the available information on relevant explanatory variables is quite limited.

Therefore, for future developments, it could be interesting to take into account some additional available information; for example, on hospitalizations, mortality data or circulating virus strains. In the present analysis we decided to not include this type of information, given two main reasons. One is to avoid some over-fitting as stated above, while another one is more conceptual: this additional information is collected in a completely different way than the ILI data; hence, its inclusion must be treated with some caution as it may require a more elaborate theoretical development. Further, another line of future research is to define the generic data sets in other ways, e.g. as partially reconstructed data from a multivariate analysis of the regions. This idea seems interesting but implies a more involved analysis that goes beyond the scope of this paper.

Finally, it is worth noting that the proposed methodology could be easily adapted for comparing incidence rates across years, in the same region. An important practical application of such a comparison would be to consider an unusual year (like a pandemic year) and try to assess its differences with regular flu seasons, in the same area, by comparing observed and predicted values. Assessing the size of a pandemic outbreak, or of an epidemic at the beginning of the flu season, are issues of great interest to epidemiologists and public health practitioners (see, e.g.^3,13,14^).

## Supplementary information

List of HHS regions

## Data Availability

The datasets analyzed during the current study are available at https://www.cdc.gov/flu/weekly/fluviewinteractive.htm.
