# Supplementary material for: A statistical tool for comparing seasonal ILI surveillance data
Source: Sci Rep. 2019 Feb 5;9:1422. doi: 10.1038/s41598-018-38292-x (PMC6363783; doi:10.1038/s41598-018-38292-x)
Supplement: Supplementary file 1 — List of HHS regions [file 41598_2018_38292_MOESM1_ESM.pdf]

Supplementary information for the paper:

A statistical tool for comparing seasonal ILI surveillance data

by René Ferland, Sorana Froda (*)

Département de mathématiques, UQAM
C.P. 8888, succ. centre-ville, Montréal, Canada, H3C 3P8

(*) Corresponding author: sorana.froda@uqam.ca

Full list of the ten HHS regions

The list below can be found on the CDC site:

`http://www.cdc.gov/flu/weekly/overview.htm.`

- **Region 1.** Connecticut, Maine, Massachusetts, New Hampshire, Rhode Island, and Vermont.
- **Region 2.** New Jersey, New York, Puerto Rico, and the U.S. Virgin Islands.
- **Region 3.** Delaware, District of Columbia, Maryland, Pennsylvania, Virginia, and West Virginia.
- **Region 4.** Alabama, Florida, Georgia, Kentucky, Mississippi, North Carolina, South Carolina, and Tennessee.
- **Region 5.** Illinois, Indiana, Michigan, Minnesota, Ohio, and Wisconsin.
- **Region 6.** Arkansas, Louisiana, New Mexico, Oklahoma, and Texas.
- **Region 7.** Iowa, Kansas, Missouri, and Nebraska.
- **Region 8.** Colorado, Montana, North Dakota, South Dakota, Utah, and Wyoming.
- **Region 9.** Arizona, California, Hawaii, and Nevada.
- **Region 10.** Alaska, Idaho, Oregon, and Washington.
